# A Cross-Sectional Study of the Perceived Stress, Well-Being and Their Relations with Work-Related Behaviours among Hong Kong School Leaders during the COVID-19 Pandemic

**DOI:** 10.3390/ijerph192315777

**Published:** 2022-11-27

**Authors:** Sam S. S. Lau, Eric N. Y. Shum, Jackie O. T. Man, Ethan T. H. Cheung, Padmore Adusei Amoah, Angela Y. M. Leung, Orkan Okan, Kevin Dadaczynski

**Affiliations:** 1 Research Centre for Environment and Human Health, School of Continuing Education, Hong Kong Baptist University, Hong Kong; 2 Multidisciplinary Research Centre, School of Continuing Education, Hong Kong Baptist University, Hong Kong; 3 College of International Education, Hong Kong Baptist University, Hong Kong; 4 Institute of Bioresource and Agriculture, Hong Kong Baptist University, Hong Kong; 5 School of Graduate Studies, Department of Applied Psychology, Institute of Policy Studies, Lingnan University, Hong Kong; 6 School of Nursing, Hong Kong Polytechnic University, Hong Kong; 7 Department of Sport and Health Sciences, Technical University Munich, 80333 Munich, Germany; 8 Public Health Centre Fulda, Fulda University of Applied Sciences, D-36039 Fulda, Germany; 9 Center for Applied Health Science, Leuphana University Lueneburg, 21335 Lueneburg, Germany

**Keywords:** COVID-19, school management, leadership, school principals, school leaders, occupational well-being, education, stress

## Abstract

The health and well-being of school leaders during the COVID-19 pandemic have been largely neglected compared to the health and well-being of students and teachers. This study assessed the magnitude of perceived stress and well-being and the associated factors, including number of working hours, work-related sense of coherence (work-SoC), perceived stress, self-endangering work behaviour, secondary burnout symptoms, and satisfaction with work, among school leaders in Hong Kong, China during the COVID-19 pandemic. This cross-sectional, survey-based study collected demographic data and mental health measurements from 259 eligible school leaders in Hong Kong from April 2021 to February 2022. Pearson’s correlation analyses, multilinear regression models, and independent-samples Student’s t-tests were performed. The findings revealed that school leaders’ perceived stress was negatively correlated with their well-being (*r* = −0.544, *p* < 0.01) and work-related SoC (*r* = −0.327, *p* < 0.01) but positively correlated with their extensification of work (*r* = 0.473, *p* < 0.01), exhaustion related to work situations (*r* = 0.559, *p* < 0.01), and psychosomatic complaints (*r* = 0.439, *p < 0*.01). In a model that adjusted for gender and age, student leaders with higher subjective well-being scores had a lower level of perceived stress (B = −0.031; 95% confidence interval [CI], −0.59, −0.02; *p* = 0.034), whereas leaders in schools with a larger student population had a higher level of perceived stress (B = 0.002; 95% CI, 0.000, 0.003; *p* = 0.030). School leaders with a higher likelihood of performing the self-endangering work behaviour of ‘intensification of work’ had higher perceived stress levels (B = 1.497; 95% CI, 0.717, 2.278; *p* < 0.001). School leaders with a higher work-related SoC (B = 4.20; 95% CI, 1.290, 7.106; *p* = 0.005) had a higher level of well-being. School leaders with higher levels of perceived stress (B = −0.734; 95% CI, −1.423, −0.044; *p* = 0.037), a higher likelihood of performing the self-endangering work behaviour of ‘extensification of work’ (B = −4.846; 95% CI, −8.543, −1.149; *p* = 0.010), and a higher score for exhaustion related to work (B = −10.449; 95% CI, −13.864, −7.033; *p* = 0.000) showed lower levels of well-being. The finding of a high incidence of stress among school leadership justifies the need for more societal attention to the well-being of school leaders in Hong Kong. It is important that policies and initiatives are designed to enhance the well-being of school leaders and that they are supported in leading the management of schools and coping with stress in school settings.

## 1. Introduction

The recent COVID-19 outbreak significantly interrupted every aspect of life [1,2]. International travel, economic growth, occupational practices, and schooling were interrupted globally. As of 1 November 2022, the COVID-19 pandemic has caused more than 6.5 million deaths, and 627 million cases have been confirmed globally [3]. During the pandemic, different forms of public health measures designed to inhibit the spread of COVID-19 were implemented around the world, including the closure of childcare centres, workplaces, public spaces, and schools. According to the United Nations Sustainable Development Group, by April 2020, approximately 1.6 billion learners in more than 190 countries encountered disrupted education due to the COVID-19 pandemic. More than 94% of the world’s student population was affected by the closure of learning spaces and schools [4]. School closures due to COVID-19 outbreaks were stressful to students [5] and teachers [6]. Studies have shown that more than 60% of educators reported high or moderately high levels of stress and an inadequate working environment during the pandemic, and the consequences of the pandemic and the increased workload were the predominant stressors during this period [6]. Even prior to the COVID-19 pandemic, the health and well-being of school leaders has been a concern, with research showing that school principals across the globe experienced work intensification [7]. Work intensification refers to an increased amount of work and effort over time along with refraining from both work breaks and social interactions at work that often results in additional stress [8,9,10]. Denecker [11] reported that 95% of school principals in Switzerland considered their job to be very stressful, and more than half felt that their occupational stress had negative effects on their health and personal life. A study of German school principals indicated low levels of work-related sense of coherence (SoC) for administrative tasks, and more than one third of the respondents reported being dissatisfied or very dissatisfied with their job [12]. The low well-being of educators worldwide is worrying, and studies are needed to assess the well-being of educators as the pandemic continues. 

### 1.1. Physical Health and Well-Being of School Leaders during the COVID-19 Pandemic

Despite the drastic effects of the COVID-19 pandemic on work and working environments, research on the health and well-being of school leaders during the COVID-19 pandemic is limited compared to research on frontline health workers [13,14]. School leaders such as principals, vice-principals, and assistant principals are central actors within schools, as they are responsible for leading all aspects of school development and making relevant decisions [12,15]. They are considered exceptional managers and excellent leaders in a time of continuous change in a digitalised environment [16]. Given the magnitude of the workload and the significance of the work, the role of school principals and leaders is highly demanding [17]. Evidence suggests that school quality and student achievement depend heavily on the quality of the school’s leadership, as improving the quality of the principal improves students’ academic performance [18,19]. The challenges of school leadership have been compounded by the COVID-19 pandemic, as school principals and leaders have been responsible for overseeing school closures and re-openings and for meeting the academic, emotional, and health needs of students and teachers who were quarantined or infected. A recent study revealed that school leaders played an important role in navigating the tensions at work during the global COVID-19 crisis, such as the tension between the well-being and the workload of students, teachers, and parents [20]. These findings suggest that school leaders are an occupational group that was under great stress and faced great challenges during the COVID-19 pandemic in Hong Kong.

### 1.2. Sense of Coherence and Burnout in School Leaders

Stress is known to have a detrimental effect on a person’s health if they lack the internal or external resources necessary to respond to requirements in a timely manner. Antonovsky [21] developed the salutogenic model, which provides the theoretical basis for the stress resource-oriented concept that focuses on resources to maintain and improve health. An SoC, which refers to individuals’ capacity to cope with life stresses and psychological distress, is a major concept within the model [21,22]. Scholars have further proposed the concept of work-related SoC, which is defined as ‘the perceived comprehensibility, manageability, and meaningfulness of an individual’s current work situation’ [23]. SoC consists of three components: comprehensibility, manageability, and meaningfulness. Comprehensibility reflects how individuals see their workplace in terms of organisation, consistency, and clarity [23]. The instrumental part of the concept is manageability, which is the extent to which an individual feels that there are sufficient resources available to satisfy the expectations of the job [24]. Finally, the motivating component determines whether or not the work environment is deemed worthy of devotion and involvement; this is referred to as meaningfulness [24]. The three components of SOC are used as personal resources that may protect individuals from stress and are also associated with a reduction of health risks [25], Individuals who have a high SOC level would see their environment as more comprehensive, manageable, and meaningful [26]. Before the pandemic, a study of German school principals indicated low levels of work-related SoC [12]. Another study of upper-secondary school teachers in Czech found that teachers with a higher SoC were better equipped to deal with adversity at work and to recognise and mobilise internal and external resources to avoid professional exhaustion and a decline in their ability to work [27]. During the pandemic, school principals were required to deal with an increased workload and stress originating from unprecedented situations [28]. An SoC may be one of the crucial factors preventing burnout in school principals. However, studies of work-related SoC among school principals during the COVID-19 pandemic have been scarce. 

### 1.3. Self-Endangering Work Behaviour in School Leaders during Pandemic

Lazarus and Folkman [29] proposed the Transactional Model of Stress and Coping, which suggests that an individual’s ability to cope with and adjust to difficulties and problems is a consequence of the transactions (or interactions) that take place between the individual and their surroundings. Based on this model, Dettmers et al. [30] proposed the concept of ‘self-endangering work behaviour’, which refers to behaviours that may be useful for achieving professional objectives but are detrimental to long-term health and productivity [30]. Examples of such behaviours are work intensification and extending work time [31]. Dettmers et al. [30] believed that self-endangering work behaviour may be seen as a type of coping reflex in response to a massive workload and increased demands for self-organisation, which negatively affect well-being. Therefore, it is worth studying the prevalence of self-endangering work behaviour and its relationship with teachers’ well-being.

### 1.4. School Leaders in Hong Kong

As in many parts of the world, the suspension of face-to-face classes at schools was part of the public health measures implemented in response to the COVID-19 pandemic in Hong Kong. It was first implemented by the Education Bureau of the Government of the Hong Kong Special Administrative Region from late January until late May 2020, during the first and second waves of the pandemic [32]. School closures and re-openings continued during the third wave and fourth waves. Schools were suspended from August 2020 until mid-late September 2020 and again from December 2020 until after the Lunar New Year holidays in 2021 [32]. Because of these school closures, schooling transitioned to online systems at an unprecedented pace. Although some principals may have experienced the severe acute respiratory syndrome coronavirus (SARS-CoV) outbreak in 2003 in Hong Kong, this rapid rate of educational reform, shifting from face-to-face learning to online learning caused by the prolonged COVID-19 pandemic, has remained a challenge and has been a stressful time for school leaders. It has been the longest period of school closures in recent decades. There were fewer than 100 school days in the whole year from 2020 to 2021, which represents a school suspension period five times longer than the suspension period that occurred during the SARS-CoV outbreak in 2003, which lasted for 51 days [33]. School leaders were required to take on the responsibility of managing the new reality in their own schools. Uncertain and ever-changing situations like those created by the COVID-19 pandemic are considered to be stressful to school leaders [34]. However, previous studies have mainly focused on the well-being and mental health status of students, teachers, and the general population during the pandemic; there is little research on school leaders’ health while they led through the pandemic [35]. Thus, there is an urgent need for empirical research on the mental health of school leaders in Hong Kong.

### 1.5. Study Objectives

Against this background, we examined the well-being of school leaders during the COVID-19 pandemic. The objectives of this study were to (1) assess the stress levels and well-being of school leaders in Hong Kong, (2) investigate the associated factors (i.e., gender, age, number of weekly working hours, teaching involvement, and position at the school) affecting the well-being of school leaders, (3) explore the differences in well-being and perceived stress levels in relation to different demographic variables, and (4) explore the level of exhaustion among school leaders in Hong Kong.

## 2. Materials and Methods

### 2.1. Study Procedures and Data Collection

This study was part of an international study on School Health Literacy based on the global COVID Health Literacy (COVID-HL) Network (https://COVID-hl.eu, accessed on 04 November 2022). The COVID-HL Network facilitates collaborative and cross-country research initiatives on health literacy and includes more than 150 researchers from more than 70 different countries. A cross-sectional descriptive study was conducted with school leaders across all schools in Hong Kong, China from April 2021 to February 2022. Eligibility was based primarily on whether the person was (1) a school leader (e.g., principal, vice-principal, assistant principal, head, member of the school management committee or incorporated management committee, dean of admissions, director of learning support, or director of operations), (2) aged over 18 years, (3) working in a school (including a primary, secondary, or special school) in Hong Kong during the pandemic, and (4) living in Hong Kong. The participants were purposively recruited via email, social media, and personal contacts. The survey was administered either as a paper-and-pencil or online questionnaire in Chinese or English. An invitation with a link to an online Qualtrics survey was disseminated via email to the leaders of 1130 schools in Hong Kong based on those registered with the Education Bureau and through the authors’ network and the social media platform WhatsApp. In addition, hardcopy questionnaires were posted to school leaders who had given prior verbal consent over the phone. The participants were informed of the objectives, duration, procedure, benefits, and risks of the study and were assured of their anonymity and confidentiality. All of the participants provided informed consent and were made aware of their right to withdraw at any time. The survey took 20–30 min to complete. The data were anonymised before being coded and evaluated. We used G*Power 3.1.9.7 software for Windows to calculate the sample size. A sample of 213 school leaders was deemed necessary based on a type I error of 0.05, a power of 0.95, an effect size of 0.1, and 18 potential predictors in the multiple linear regressions.

### 2.2. Instruments and Measurements

The questionnaires assessed demographics, job-related aspects, work-related SoC, perceived stress, self-endangering behaviour, work satisfaction, and health-related information in the context of the COVID-19 pandemic.

#### 2.2.1. Demographic Indicators

Data for the following sociodemographic variables were collected: gender (male vs. female), age (year), type of school (primary, secondary, or special), and position at the school (school principal, school vice-principal, school assistant principal, or member of the school management committee or leadership team).

#### 2.2.2. Work-Related Factors

The participants were asked about their weekly number of teaching hours, weekly number of working hours, changes in the number of weekly working hours as a consequence of the COVID-19 pandemic, the number of students in their schools, and the approximate percentages of students with low, middle, and high socioeconomic statuses.

#### 2.2.3. Well-Being

The well-being of the respondents was assessed using the 5-item World Health Organization Well-being Index (WHO-5) [36]. The respondents were asked to rate how often they had experienced the feelings described in the statements over the last 2 weeks (e.g., ‘I have felt cheerful and in good spirits’; ‘I have felt calm and relaxed’; ‘I woke up feeling fresh and rested’). Responses were scored on a 6-point scale ranging from 0, indicating ‘never’, to 5, indicating ‘always’. A total raw score ranging from 0 to 25 was calculated, and was then multiplied by 4 to obtain the final score. Scores of 0 and 100 represented the worst and best levels of well-being, respectively. This score can be used to screen for depression, with a recommended cut-off score less than or equal to 50 indicating poor well-being and a score of 28 or lower indicating depression [37].

#### 2.2.4. Exhaustion and Psychological Discomfort

To assess exhaustion related to work situations, we used the ‘exhaustion’ dimension of the Burnout Assessment Tool (BAT) [38]. All items were scored on a five-point Likert scale ranging from 1 (‘never’) to 5 (‘always’). Responses were summed and divided by the number of items, with higher score indicating a greater level of exhaustion. The ‘exhaustion’ dimension has been shown to have satisfactory reliability [39].

To measure self-reported psychological discomfort, we used the five-item ‘psychosomatic complaints’ dimension of the BAT [39]. Each item is rated on a five-point Likert scale from 1 (‘never’) to 5 (‘always’). Responses were summed and dividing by the number of items, with higher score indicating more psychosomatic complaints.

As recommended by Schaufeli, De Witte and Desart [38], the total scores for exhaust dimension and psychosomatic complaints were classified into four levels, ‘low’, ‘average’, ‘high’, and ‘very high’.

#### 2.2.5. Work-Related Sense of Coherence

SoC is the main component of Antonovsky’s salutogenesis theory. Salutogenesis focuses on health resources, asking the question of what makes people healthy [22]. Based on the studies of Antonovsky and other researchers around the world, the answer to that question is SoC [22,40,41,42,43]. SoC, as a general life orientation, helps people to acquire and use health resources to manage their stressors and promote their health and well-being. In the present study, SoC was assessed using a self-reported 9-item scale within the work context [44]. After giving an introduction (‘How do you personally find your current job and work situation in general?’), nine bipolar adjective pairs were presented that could be rated on a 7-point scale. Three subscales involving comprehensibility, manageability, and meaningfulness were included in the scale. Comprehensibility was measured by items 1, 3, 6, and 9, which included ‘manageable–unmanageable’ assessments. Manageability was measured by items 4 and 7, which included ‘controllable–uncontrollable’ assessments. Items 2, 5, and 8 measured meaningfulness using ‘significant–insignificant’ assessments. The mean scores for the items were calculated as the total scores, with higher scores indicating a higher SoC. This scale has been shown to have very good reliability (Cronbach’s α = 0.83) and acceptable to good reliability within subscales (Cronbach’s α = 0.72 to 0.84) [23].

#### 2.2.6. Perceived Stress

The 10-item Perceived Stress Scale [45] was used in the context of the school setting and COVID-19 to measure work-related stress. It contains two subscales, perceived helplessness and perceived self-efficacy. Perceived helplessness was measured by items 1, 2, 3, 6, 9, and 10, which included the following question: ‘How often have you been upset because of something that happened unexpectedly at your work at school?’. Items 4, 5, 7, and 8 were used to measure perceived self-efficacy, and included the following question: ‘How often have you been able to control irritations at your work at school?’. Participants rated these items on a 5-point scale from 1 = ‘never’ to 5 = ‘very often’. After reversing the scoring for items 4, 5, 7, and 8, the sum of all 10 items was calculated as the total score. A higher total score indicated a greater level of perceived stress by the respondent. This scale has been shown to have satisfactory reliability (perceived helplessness: Cronbach’s α = 0.85; perceived self-efficacy: Cronbach’s α = 0.88–0.89), and confirmatory factor analysis and correlation analyses supported high content validity and construct validity [46].

#### 2.2.7. Self-Endangering Work Behaviour

To assess self-endangering behaviours, we used three subscales of the subjective self-endangering work behaviour scale [47], including six-item ‘extensification of work’, three-item ‘intensification of work’, and three-item ‘quality reduction’ (e.g., ‘... give up leisure activities in favour of work’, ‘...you find burdensome?’, and ‘...satisfied even with a worse work result than you would normally be?’). All items were scored on a 5-point Likert scale that ranges from 1 (‘never/very rarely’) to 5 (‘very often’). The reliability and validity have been reported previously [47].

#### 2.2.8. Work Satisfaction

Work satisfaction was assessed by asking the respondents to rate how satisfied they were with their overall work using a 5-point scale ranging from 1, indicating ‘very dissatisfied’, to 5, indicating ‘very satisfied’.

### 2.3. Data Analysis

The collected data were analysed using Statistical Package for Social Science version 27.0 for Windows (IBM Corp., Armonk, NY, USA). Continuous data are presented as means (M) with standard deviations (SD). Categorical data are presented as counts (*n*) and relative frequencies (%). Correlation and linear regression analyses were performed to explore the correlations between perceived stress, well-being, and other outcome variables. Independent-samples Student’s t-tests were performed to explore the mean differences in other independent variables between the groups with high and low levels of perceived stress. A *p*-value less than 0.05 was considered statistically significant.

### 2.4. Ethical Considerations

This research was conducted with the approval of the Research Ethics Committee of Hong Kong Baptist University, Hong Kong, China (REC/20-21/0465). All of the school leaders participated in the study anonymously and voluntarily. No personal data were collected. They were informed of the study’s objectives and the collection, anonymisation, and privacy of all personal data. All participants provided informed consent before their inclusion in the study.

## 3. Results

Table 1 shows the demographic and work characteristics of the participants.

### 3.1. Characteristics of the Participants

Two hundred and fifty-nine school leaders responded to the survey. The mean age of the respondents was 48.6 years (SD = 7.48), ranging from 22 to 68 years, with slightly more than half of the participants were male (56.4%). 64.9% of the respondents were involved in teaching. The majority of the respondents (67.3%) worked at secondary schools, 25.7% worked at primary schools, and 7.0% worked at special schools. The average number of working hours per week was 52.1 ± 15.73, which is considerably higher than the population average of 44 h per week [48]. More than half of the respondents (54.6%) worked more than 50 h per week, and 42.1% of the respondents worked more during than before the COVID-19 pandemic (Table 1).

### 3.2. Number and Socioeconomic Status of the Students

Most (64.5%) of the respondents reported that the student population at their school range from 600 to 1000, 31.5% reported a student population less than or equal to 600, and 3.9% reported a student population greater than or equal to 1000. The average percentages of students from families with lower, middle, and higher socioeconomic statuses were 46.4 ± 25.8%, 38.8 ± 20.9%, and 11.9 ± 15.0%, respectively.

### 3.3. Work-Related Sense of Coherence

The mean work-related SoC score was 4.91 ± 0.77. The mean scores for the three subscales of comprehensibility, manageability, and meaningfulness were 4.85 ± 0.97, 4.22 ± 0.97, and 5.46 ± 1.04, respectively.

### 3.4. Perceived Stress

The mean score for perceived stress was 31.1 ± 3.90. Most of the respondents (90.1%) were categorised in the high-stress category, with 23 respondents (9.1%) in the moderate-stress category and none in the low-stress category.

### 3.5. Self-Endangering Work Behaviours

The mean scores for extensification of work, intensification of work, and quality reduction were 3.61 ± 0.75, 3.07 ± 0.85, and 2.51 ± 0.76, respectively.

### 3.6. Work Satisfaction

Almost half of the respondents (48.2%) were very satisfied or quite satisfied with their work. Only 32 respondents (12.6%) were very dissatisfied or quite dissatisfied with their work. The remaining respondents (39.2%) were neither satisfied nor dissatisfied with their work.

### 3.7. Well-Being

The mean WHO-5 index score of the respondents was 54.0 ± 20.3. For exhaustion related to work situations, the percentages of respondents classified as ‘very high’, ‘high’, ‘average’, and ‘low’ were 26.3%, 36.2%, 35.5%, and 2.0%, respectively. For psychosomatic complaints, the percentages of respondents classified as ‘very high’, ‘high’, ‘average’, and ‘low’ were 17.2%, 57.0%, 21.1%, and 4.7%, respectively.

Table 2 presents the interrelations between the key variables. A strong positive correlation was found between perceived stress and the intensification of work. Perceived stress was moderately negatively correlated with the WHO-5 well-being index score and work-related SoC and moderately positively correlated with the extensification of work, exhaustion related to work situations, and psychosomatic complaints. The WHO-5 well-being index score was strongly or moderately negatively correlated with exhaustion related to work, the intensification of work, the extensification of work, and psychosomatic complaints.

### 3.8. Factors Associated with Perceived Stress

Multiple linear regression models adjusted for age, gender, and position at the school were computed to explore the associations between perceived levels of stress and other independent variables. Table 3 shows that higher perceived stress levels were associated with (1) work as a vice-principal (B = 1.149; 95% confidence interval [CI], 0.105, 2.193; *p* = 0.031), (2) higher ‘intensification of work’ scores in the self-endangering work behaviour scale (B = 0.182; 95% CI, −0.577, 0.940; *p* = 0.001), and (3) higher scores in the ‘exhaustion related to work’ dimension (B = 0.568; 95% CI, −0.063, 1.199; *p* = 0.002; *F* [16,188] = 11.386; *p* < 0.001).

Table 4 provides the results of independent-samples Student’s t-tests and least significant difference post hoc tests, which were used to compare the perceived stress scores and well-being index values by gender and position at the school. Female leaders attained higher scores (31.21 ± 3.61) than male leaders (30.86 ± 3.80) for perceived level of stress, although no significant difference was found (*t* [253] = −0.756, *p* = 0.450). Assistant principals (32.14 ± 4.41) reported higher scores than school principals (30.75 ± 3.57), school vice-principals (31.21 ± 3.70), members of school management committees (29.40 ± 2.01), and leadership team members (31.59 ± 5.03) for perceived level of stress, although there were no significant differences found between positions at the school. There were no significant differences in perceived levels of stress between the three age groups.

Male leaders reported higher well-being index scores (55.75 ± 19.31) than female leaders (52.14 ± 20.97), but the difference was not statistically significant (*t* [256] = 1.434, *p* = 0.136). Participants who worked as school assistant principals (48.72 ± 20.35) reported significantly lower well-being index scores than those who worked as members of school management committees (64.80 ± 23.00). There were no other statistically significant differences in the level of well-being between the remaining position groups in our study. There were no significant differences in the level of well-being among the three age groups.

### 3.9. Factors Associated with Well-Being

Multiple linear regression models adjusted for age, gender, and position at the school were used to explore the associations between the level of well-being and the other independent variables. Table 5 presents the regression coefficients for predicting the level of well-being based on other factors. Higher WHO-5 index scores were associated with (1) lower ‘exhaustion related to work’ subscale scores (B = −11.784, 95% CI, −15.366, −8.203; *p* < 0.001) in the self-endangering work behaviour scale, (2) lower ‘extensification of work’ subscale scores in the self-endangering work behaviour scale (B = −4.201; 95% CI, −7.975, −0.427; *p* = 0.029), and (3) higher work-related SoC (B = 4.466; 95% CI, 1.513, 7.419; *p* = 0.003; *F* [19,189] = 12.72, *p* < 0.001). There were no significant associations between the level of well-being and the remaining independent variables.

## 4. Discussion

The present study revealed a high perceived stress level in school leaders in Hong Kong, with the vast majority of participants (90.1%) categorised as highly stressed. The perceived stress score (31.1 ± 3.89) of school leaders in Hong Kong was remarkably higher than the scores reported elsewhere (e.g., 12.7 ± 4.5 in Taiwan, Duong et al. [49]). This is consistent with the findings of Reid [50], who found that school leaders in the U.S. reported increased levels of stress during the COVID-19 pandemic. Other studies have also shown that school staff have encountered mental health difficulties and challenges since the onset of the pandemic [51,52,53,54].

Our findings suggest that school leaders’ perceived levels of stress vary with different demographic factors. The perceived level of stress was slightly higher in female than male school leaders, although the difference was not significant. This finding may be explained by the fact that females are generally more involved in caring functions at home, resulting in additional responsibilities and, hence, more stress [55]. Our findings support those of Santomauro et al. [56], whose systematic review of studies from 204 countries and territories found that females were generally more affected than males by depressive disorders during the COVID-19 pandemic. However, previous studies on the gender difference in the stress levels of school principals have found mixed results. Although some studies have shown that male principals experienced slightly higher stress levels compared to their female counterparts [57,58,59], other studies found that females experienced higher levels of stress than males in educational settings [60,61].

Our results showed no significant association between participants’ perceived level of stress and their age. However, a recent study reported that school principals’ stress level is inversely correlated with their age [62]. Further research has suggested that the level of perceived stress varies with the experience of the school principal, as those with less experience had a higher level of perceived stress than more senior principals [57]. However, a previous study of primary and high-school teachers in Greece found that older teachers reported higher levels of stress related to the support they perceived to be receiving from the government, whereas younger teachers reported higher levels of burnout, as evidenced by emotional exhaustion and disengagement from the profession [63]. These previous findings demonstrated that age may affect the perceived stress of teaching and administrative staff, and this may depend on the context of the stressor. Thus, the association between principals’ perceived stress and age remains unclear under the context of COVID-19. Whilst it is expected that younger principals may not have a frame of reference from past experiences, this disadvantage may not be applicable to the COVID-19 pandemic, as it is the first time in half a century that school principals have faced such a large-scale prolonged pandemic. At the same time, older principals may not benefit from their experience and wisdom, as they have no comparable experience. Thus, future studies may focus on the stresses that principals of different ages experience as the pandemic continues.

The association between higher ‘intensification of work’ subscale scores and self-endangering work behaviour scales and higher levels of perceived stress in our study echo the findings of several previous studies showing that work intensification is one of the critical work stressors regardless of the occupation [64,65,66]. The present study demonstrated the mutual relationship between work intensification and perceived stress among school principals. Previous studies revealed that increasing work intensification causes stress to employees [67,68]. These findings are consistent with the concept proposed by the Transactional Model of Stress and Coping [29,30], in which self-endangering work behaviour is the transaction (or interaction) that occurs between individuals and their environment when the individuals are trying to find resources [30]. In the current study, in an adjusted regression model, perceived stress predicted self-endangering work behaviour (i.e., work intensification). Thus, predicting self-endangering work behaviour by school leaders based on their perceived level of stress is worth investigating in the future.

Our study revealed that work intensification was strongly correlated with work-related exhaustion, which is in line with the findings of Boxall and Macky [67]. Previous studies have found that emerging policies and changes in the education system were sources of increasing work intensification pressure on school management staff [69,70]. Under the context of the COVID-19 pandemic, emerging policies and changes in the delivery of education, such as the adoption of online learning, often occurred in response to the severity of the pandemic [71]. Such changes may have varied the nature of educational leadership work, such that school leaders needed to adopt new methods to communicate with students and their families and deal with disciplinary problems in new forms of education, such as attendance and appearance in online settings [72].

In agreement with other studies, we found that a greater number of weekly working hours was associated with higher levels of perceived stress. It has long been documented that longer working hours are associated with higher levels of stress and lower quality of life [73,74,75]. A previous study in Korea suggested that those who worked more than 50 h per week were more likely to report high levels of perceived stress [76]. Long working hours have been associated with the psychosocial stress responses of white-collar workers [77]. In the context of COVID-19, an increase in the number of weekly working hours was associated with increased perceived stress levels among nursing staff in Austria [75]. Studies have also shown a significant increase in the number of teachers’ working hours since October 2020 [78], and teachers may have experienced burnout during this period of crisis [79,80]. There is a lack of literature concerning the perceived stress of school leaders in relation to the number of working hours during the pandemic. However, it has been documented that the diverse range of expectations from schools and educational reforms have increased the workload and the level of stress for principals worldwide [12,81,82]. Indeed, it is inevitable that the numerous COVID-19-related control measures, which have affected the organisation of schools and the teaching and learning structure in many ways, also affected the work schedule and workload of school personnel. The effects of such changes on the stress levels of school personnel are therefore understandable.

Vice principals and assistant principals tended to have a higher level of perceived stress than principals. Vice principals play a crucial mediating role between teachers, students, principals, and the school organisation [83]. Recent studies have suggested that more than 70% of vice principals in Japan, as intermediaries between staff teachers and their principals, who work overtime for 3 h or more each day, may be under more role stress than staff teachers and principals [84,85]. In addition, the widespread educational reforms, which have contributed to the expanded responsibilities of vice principals, may also explain their higher stress levels [86]. In Hong Kong, Walker and Kwan [87] reported that vice principals who did not aspire to become principals experienced higher levels of stress than those who did have such aspirations. Thus, additional attention should be placed on the mental health of principals, especially vice principals and assistant principals.

In the current study, lower ‘exhaustion related to work’ scores were associated with greater well-being. Our findings replicate those of a previous study showing that perceived stress in school principals increases burnout, in turn, affecting their well-being [88]. Moreover, it has been documented that experiencing work fatigue is one of the vital issues in educators’ well-being [89]. Our findings indicated that exhaustion related to work affected leaders’ well-being during the pandemic. In the context of the widespread well-being crisis during the COVID-19 pandemic, health authorities and policy makers need to review the healthcare system to provide support for leaders’ well-being. In addition, we found that lower ‘extensification of work’ scores were associated with greater well-being. This is in line with a previous study showing that increased job intensity increases the risk of burnout and stress in employees, and contributes to poor work–life balance [67]. Moreover, higher SoC scores and better perceived general health predicted greater well-being in our study.

Significant association was found between well-being and perceived stress in regression models. However, a moderate negative association between perceived stress and work-related SoC was identified. In a previous study of leadership professions, high levels of stress were found to affect the leaders’ well-being [90]. However, research suggests that the workload and role balance, not the school or individual qualities, may explain principals’ levels of well-being [91]. Support from education sector organisations and government agencies, stressors from parents and employees, the principal’s fitness level, and engagement in principal networks have all been shown to be major contributors to well-being [92]. Therefore, the well-being of school leaders is a multi-dimensional concept, influenced by multiple factors.

## 5. Strengths and Limitations

The research described in this paper helps fill a research gap in the effects of the COVID-19 pandemic on the mental health and well-being of school leaders. One key strength of the study is the relatively heterogeneous sample that allowed us to explore different subgroups (e.g., principals, vice principals, and assistant principals). However, some limitations should be considered when interpreting the results. First, we employed a cross-sectional design, such that it was not possible to prove causality. Second, as with any self-report survey-based study, response bias may have affected the validity of our findings. Social desirability bias may lead to the over-reporting of positive factors or the under-reporting of negative factors. Third, although invitations were extended via a purposive sampling approach, initially to all schools in Hong Kong, the study population may not be representative of all school leaders as additional efforts were made to reach out to participants through the authors’ personal networks to increase the response rate. This may have limited the generalisability of the findings to the broader school leader population. Fourth, as the majority of the participants in the study were categorised in the high-stress group, there may have been selection bias, in that the school leaders who felt stressed were more likely to participate in the study. The significance of the sampling approach in mental health studies has been noted as a key concern during COVID-19 [93]. Fifth, the study was limited by the data collection difficulties and thus the extended data collection period (i.e., 11 months) during the COVID-19 outbreaks in Hong Kong. During which, school operations varied between half-day face-to-face lessons and suspending face-to-face classes. Finally, our study was based on a quantitative approach only, and we did not assess mental well-being using qualitative methods. Further studies using a longitudinal mixed methods design and a larger, more representative sample would be of value. In addition, future research in developing moderated or mediated models (e.g. [94]) in examining whether different variables may moderate or mediate the relationship between stress and wellbeing is warranted.

## 6. Conclusions

Recognising the profound and enduring effects of the lingering COVID-19 pandemic on mental health [95], rigorous empirical evidence is critical to improve our understanding of its effects in school settings. Previous research in school environments has tended to concentrate on the mental health and well-being of students (e.g., Drane et al. [96]) and teachers (e.g., Fray et al. [97]), rather than school leadership. The present study contributes to the scant literature on the mental health and well-being of school leaders during the COVID-19 pandemic, especially in China. School leaders were already subjected to stress prior to the pandemic due to their leadership role, and the results of the current study underscore the need to find ways to support school leaders to maintain their mental health during times of crisis. The findings presented herein highlight the need for policies and initiatives aimed at enhancing the mental health and well-being of school leaders in Hong Kong school settings during the pandemic and beyond. A practical implication for school management emerging from this study is to create conditions for a support system, in which school leaders are included, to provide mental health and well-being support. We hope that our study encourages school leadership community and the Education Bureau to discuss strategies and ways to better support school leaders to help them better promote health and wellbeing. With the ongoing ramifications of the COVID-19 pandemic, it is essential that additional efforts are paid to the continued monitoring of school leaders’ mental health and well-being.

## Figures and Tables

**Table 1:** Demographic characteristics, school types and situations, perceived stress, health, self-endangering work behaviours, work-related SoC, and well-being of school leaders (*n* = 259).

Variables		*n* (%)
Age, mean ± SD, 22–68 years		48.6 ± 7.48
Age group	45 or below	31.2 ± 4.94
	46 to 50	31.0 ± 3.36
	51 or above	31.1 ± 3.51
Gender	Male	146 (56.6)
	Female	112 (43.4)
School type	Primary school	66 (25.7)
	Secondary school	173 (67.3)
	Special school	18 (7.0)
Current position at school	School principal or head of school	210 (81.8)
	Member of the school management committee or incorporated management committee	10 (3.9)
	Leadership team (e.g., dean of admissions, director of learning support, director of operations)	39 (15.1)
Involved in teaching	Yes	163 (64.9)
	No	88 (35.1)
Weekly number of working hours, mean ± SD		52.1 ± 15.7
Change in weekly number of working hours during the COVID-19 pandemic	Lower than before the COVID-19 pandemic	16 (6.2)
	About the same	134 (51.7)
	Higher than before the COVID-19 pandemic	109 (42.1)
Number of students at school, mean ± SD		630.4 ± 246.7
Number of students at school	≤600	80 (32.0)
	601–1000	170 (65.6)
	≥1001	6 (2.3)
Work-related SoC, mean ± SD		4.91 ± 0.77
Perceived stress, mean ± SD		31.1 ± 3.89
Self-endangering behaviours, mean ± SD	Extensification of work	3.61 ± 0.75
	Intensification of work	3.07 ± 0.85
	Quality reduction	2.51 ± 0.76
Work satisfaction	Very dissatisfied/Quite dissatisfied	32 (12.6)
	Neither satisfied nor dissatisfied	100 (39.2)
	Very satisfied/Quite satisfied	123 (48.2)
Exhaustion related to work situation, Burnout Assessment Tool	Very high	82 (31.7)
	High	80 (30.9)
	Average	88 (34.0)
	Low	9 (3.5)
Psychosomatic complaints	Very high	12 (4.7)
	High	54 (21.1)
	Average	146 (57.0)
	Low	44 (17.2)

SD, standard deviation; SoC, sense of coherence.

**Table 2:** Intercorrelations between key variables.

Variables	1	2	3	4	5	6	7	8	9	10	11	12
**1. Age**	-	0.117	−0.244 **	0.092	0.109	0.152 *	0.052	0.026	0.105	−0.001	−0.029	−0.069
**2. Weekly number of working hours**		-	−0.133 *	0.144 *	0.065	−0.056	0.081	−0.103	0.324 **	0.088	0.038	0.087
**3. Weekly number of teaching hours**			-	−0.047	0.000	0.005	−0.097	0.058	−0.102	−0.052	−0.065	−0.092
**4. Number of students**				-	0.067	−0.180 **	0.178 **	−0.134 *	0.321 **	0.371 **	0.051	0.200 **
**5. Work-related SoC**					-	−0.327 **	0.397 **	−0.218 **	−0.389 **	−0.241 **	−0.372 **	−0.268 **
**6. Perceived stress**						-	−0.544 **	0.473 **	0.621 **	0.299 **	0.595 **	0.439 **
**7. WHO-5 Well-Being Index score**							-	−0.458 **	−0.554 **	−0.189 **	−0.706 **	−0.471 **
**8. Self-endangering behaviour:** **Extensification of work**								-	0.654 **	0.129 *	0.459 **	0.331 **
**9. Self-endangering behaviour:** **Intensification of work**									-	0.353 **	0.667 **	0.516 **
**10. Self-endangering behaviour:** **Quality reduction**										-	0.265 **	0.219 **
**11. Exhaustion related to work**											-	0.559 **
**12. Psychosomatic complaints**												-

** *p* < 0.01; * *p* < 0.05; SoC, sense of coherence.

**Table 3:** Factors associated with the level of perceived stress via multiple linear regression analyses.

Variables	B (95% CI)	β	*p*
Gender	−0.393 (−1.220, 0.434)	−0.051	0.350
Age	−0.003 (−0.063, 0.057)	−0.006	0.920
Position (vice-principal)	1.149 (0.105, 2.193)	0.141	0.031 *
Position (school assistant principal)	1.186 (−0.345, 2.717)	0.093	0.128
Position (school management committee)	−0.125 (−2.445, 2.195)	−0.006	0.915
Position (leadership team member)	−0.074 (−1.552, 1.403)	−0.007	0.921
Weekly number of teaching hours	−0.016 (−0.057, 0.025)	−0.046	0.448
Weekly number of working hours	0.030 (0.002, 0.058)	0.126	0.035
Number of students	0.001 (0.000, 0.003)	0.104	0.067
Work-related sense of coherence	0.004 (−0.531, 0.674)	0.001	0.990
Self-endangering behaviour: Extensification of work	0.219 (−0.593, 0.600)	0.035	0.637
Self-endangering behaviour: Intensification of work	0.182 (−0.577, 0.940)	0.308	0.001 *
Self-endangering behaviour: Quality reduction	1.416 (0.614, 2.218)	0.103	0.078
Exhaustion related to work	0.568 (−0.063, 1.199)	0.252	0.002 *
Psychosomatic complaints	1.151 (0.429, 1.872)	0.133	0.044
Percentage of students in school from families with a lower socioeconomic status	0.802 (0.021, 1.582)	0.040	0.463
R-squared value	0.492		

* *p* < 0.05; CI, confidence interval; B, the unstandardized beta; β, the standardized beta.

**Table 4:** Results of independent-samples Student’s t-tests and least significant difference post hoc tests.

Grouping Variables	Grouping Variables	Perceived Stress			Well-Being Index (WHO-5) Score		
		Mean Diff.	Sig. Error	*p*	Mean Diff.	Sig. Error	*p*
Male	Female	−0.35511	0.46979	0.450	3.61057	2.51853	0.153
Principals	School vice-principals	−0.46671	0.57139	0.415	1.76311	2.97486	0.554
	School assistant principals	−1.38879	0.91284	0.129	5.83583	4.76806	0.222
	Members of the school management committee	1.34757	1.28733	0.296	−10.23689	6.72414	0.129
	Leadership team	−0.84702	0.74493	0.257	−0.61638	3.81689	0.872
School vice-principals	School assistant principals	−0.92208	0.93083	0.323	4.07273	4.85610	0.402
	Members of the school management committee	1.81429	1.30015	0.164	−12.00000	6.78686	0.078
	Leadership team	−0.38031	0.76687	0.620	−2.37949	3.92632	0.545
School assistant principals	Members of the school management committee	2.73636	1.48229	0.066	−16.07273 *	7.74248	0.039 *
	Leadership team	0.54177	1.04636	0.605	−6.45221	5.41299	0.234
Members of the school management committee	Leadership team	−2.19459	1.38521	0.114	9.62051	7.19585	0.182
Age Group: 45 or below	Age Group: 46 to 50	0.27891	0.68478	0.684	2.45591	3.50335	0.484
	Age Group: 51 or above	0.11469	0.59215	0.847	−1.30526	3.03458	0.667
Age Group: 46 to 50	Age Group: 51 or above	0.16422	0.62912	0.794	−3.76118	3.22076	0.244

* *p* < 0.05.

**Table 5:** Factors associated with the level of well-being via multiple linear regression analyses.

Variables	B (95% CI)	β	*p*
Gender	0.476 (−0.231, 0.369)	0.012	0.819
Age	0.069 (−3.631, 4.583)	0.026	0.651
Position (vice-principal)	−0.786 (−6.009, 4.437)	−0.019	0.767
Position (school assistant principal)	−1.241 (−8.904, 6.421)	−0.019	0.750
Position (school management committee)	10.463 (−1.143, 22.069)	0.096	0.077
Position (leadership team member)	7.041 (−0.312, 14.394)	0.129	0.060
Weekly number of teaching hours	−0.074 (−0.279, 0.131)	−0.041	0.479
Weekly number of working hours	0.038 (−0.102, 0.179)	0.031	0.590
Number of students	0.001 (−0.007, 0.009)	0.009	0.864
Work-related sense of coherence	4.466 (1.513, 7.419)	0.177	0.003 *
Self-endangering behaviour: Extensification of work	−4.201 (−7.975, −0.427)	−0.157	0.029 *
Self-endangering behaviour: Intensification of work	0.063 (−3.895, 4.021)	0.003	0.975
Self-endangering behaviour: Quality reduction	0.585 (−2.545, 3.716)	0.021	0.713
Exhaustion related to work	−11.784 (−15.366, −8.203)	−0.494	0.000 *
Psychosomatic complaints	−2.115 (−5.956, 1.727)	−0.068	0.279
Percentage of students in school from families with a lower socioeconomic status	−0.056 (−0.135, 0.022)	−0.073	0.160

* p < 0.05.

## Data Availability

The data that support the findings of this study are available from the corresponding author upon reasonable request.
